# Molecular detection and characterization of three novel parvoviruses belonging to two different subfamilies in zoo birds

**DOI:** 10.21203/rs.3.rs-2593815/v1

**Published:** 2023-03-07

**Authors:** Yan Wang, Yijie Sun, Xin Li, Rong Chen, Wang Li, Li Ji, Qifan Zhao, Likai Ji, Shixing Yang, Wen Zhang

**Affiliations:** Jiangsu University School of Medicine; Jiangsu University School of Medicine; The Affiliated Taizhou People’s Hospital of Nanjing Medical University; Nanjing Hongshan Forest Zoo; The Affiliated Taizhou People’s Hospital of Nanjing Medical University; Jiangsu University School of Medicine; Jiangsu University School of Medicine; Jiangsu University School of Medicine; Jiangsu University; Jiangsu University School of Medicine

## Abstract

Birds carry a large number of viruses that may cause diseases in animals or human. At present, virome of zoo birds are limited. In this study, using viral metagenomics method, we investigated the feces virome of zoo birds collected from a zoo of Nanjing, Jiangsu Province, China. Three novel parvoviruses were obtained and characterized. The genome of the three viruses are 5,909 bp, 4,411 bp and 4,233 bp in length respectively which encoded four or five ORFs. Phylogenetic analysis indicated that these three novel parvoviruses clustered with other strains formed three different clades. Pairwise comparison of NS1 amino acid sequences showed that Bir-01–1 shared 44.30%−74.92% aa sequence identity with other parvoviruses belonging to the genus *Aveparvovirus*, while Bir-03-1 and Bir-04–1 had lower than 66.87% and 53.09% aa sequence identity with other parvoviruses belonging to the genus *Chaphamaparvovirus*. These three viruses were identified as three novel species of the genus *Aveparvovirus* and *Chaphamaparvovirus* respectively basing on the species demarcation criteria of parvovirus. Our findings broaden the knowledge of the genetic diversity of parvovirus and provide epidemiological data for the outbreak of potential bird’s parvovirus disease.

Members of the family *Parvoviridae* are small, non-enveloped, icosahedral viruses with single-stranded linear DNA genome of about 3.9–6.3 kb in length which mainly encode two proteins including a nonstructural replicase protein (NS1) and a capsid (VP1) protein. Parvoviruses as ancient viruses can infect various animals and cause illness either alone or with other viruses. At present, parvoviruses are classified into three subfamilies based on the revised taxonomy: *Parvovirinae* (10 genera), *Densovirinae* (8 genera), and *Hamaparvovirinae* (5 genera) [1]. Viruses within the subfamily *Parvovirinae* infect mammals, birds, and reptiles, members of the subfamily *Densovirinae* mainly infect invertebrate hosts including insects, crustacean, and echinoderms. Members in *Hamaparvovirinae* can be detected in both vertebrate and invertebrate hosts. It’s a newly established subfamily which not only includes 3 genera in the previous *Densovirinae* but also the formerly unclassified “chapparvoviruses” in *Parvovirinae*. Among the ten genera of the *Parvovirinae* subfamily, species from *Dependoparvovirus* and *Aveparvovirus* genera have been reported in avian. Members of *Dependoparvovirus* like goose parvovirus (GPV) and muscovy duck parvovirus (MDPV) can cause fatal disease of goslings and Muscovy ducklings [2, 3]. Viruses belonging to *Aveparvovirus* genus such as turkey parvovirus (TuPV) and chicken parvovirus (ChPV) are highly infectious in young poultry, but are not sure if associated with avian diseases [4]. *The Aveparvovirus* genus is divided into three species including Columbid aveparvovirus 1, Galliform aveparvovirus 1, and Gruiform aveparvovirus 1. Many aveparvovirus-like viruses were recently detected in various wild birds [5, 6]. It hinted that some novel species of the genus *Aveparvovirus* need to be further divided.

In 2018, cloacal swabs of 58 birds of 14 species from 3 orders (*Gruiformes*, *Galliformes*, *Psittaciformes*) were collected from a zoo in Nanjing city in Jiangsu province (Table S1). All cloacal swabs were collected using disposable absorbent cotton swabs to be stored and shipped to laboratory on dry ice. Sample preparation for metagenomic sequencing was performed as previously described. The swab tips were immersed into 500 uL Dulbecco phosphate buffered saline (DPBS) and vortexed vigorously for 10 minutes. The supernatants were then collected in new 1.5ml Eppendorf tubes and centrifuged at 15,000 × g for 10 minutes and stored at −80°C for standby. For viral metagenomic sequencing, four libraries were constructed based on sampling number. A 50 μL aliquot of the supernatant was removed from each tube and collected to form four pools (700–750 μL supernatant for one pool). Sample pools were mixed and centrifuged at 12,000 × g for 20 min at 4°C. The supernatant was filtered through 0.45 μm filter (Merck Millipore, MA, USA) to remove eukaryotic and bacterial cell sized particles. Filtrates were then digested using DNase and RNase at 37°C for 60 min. Total nucleic acids were then extracted using QIAamp MinElute Virus Spin Kit (Qiagen, HQ, Germany) according to the manufacturer’s protocol.

The enriched viral nucleic acids from each pool were individually subjected to reverse transcription reactions using reverse transcriptase with 6 base random primers, then a single round of DNA synthesis using Klenow fragment polymerase (New England BioLabs, MA, USA) was performed. Four libraries were constructed using the Nextera XT DNA Sample Preparation Kit (Illumina, CA, USA) and sequenced on Miseq Illumina platform with 250 bases paired ends with dual barcoding for each pool. For bioinformatic analysis, barcodes of raw sequences were clipped using the vendor software of Illumina. An in-house analysis pipeline running on a 32-node Linux cluster was used to process the data. Reads were considered duplicates if bases 5 to 55 were identical and only one random copy was kept. Low sequencing quality tails were trimmed using Phred quality score 10 as the threshold. The total read number of each library is shown in Table 1. The adapter is trimmed using NCBI VecScreen (http://www.ncbi.nlm.nih.gov/tools/vecscreen/) with special parameters designed for adapter removal. Bacterial reads were subtracted by mapping to the bacterial blast NT database using bowtie2 v2.2.4. The cleaned reads were *denovo* assembled by SOAPdenovo2 r240 using kmer size 63 and default settings. The assembled contigs, along with singlets were aligned with an in-house viral proteome database using BLASTx (v.2.2.7) with E-value cut-off of < 10 ^−5^, where the viral BLASTx database was compiled using the fasta file of NCBI virus reference proteome (https://ftp.ncbi.nih.gov/refseq/release/viral/) (based on the annotation classification in the virus Kingdom).

A total of 9,099,916 reads were obtained from 4 libraries. Contigs and singlets were matched against a customized viral proteome database using BLASTx with an E value cutoff of < 10^−5^. Of those, 6,829 reads were assigned to the family of *Parvoviridae*. Three complete genome of parvoviruses were obtained by assembling and PCR to bridge sequence gaps, which were named Bir-01–1 (mean coverage 1,134), Bir-03-1 (mean coverage 25) and Bir-04–1 (mean coverage 126.7). The three parvoviruses were deposited in the GenBank database with accession numbers OP894050 to OP894052. The raw sequence reads from the metagenomic library were deposited in the Shirt Read Archive of the GenBank database (Table S1).

The genomes of these three viruses are 5,909 bp, 4,411 bp and 4,233 bp in length, respectively. The ORF were predicted using a combination of Geneious 11.1.2 software and NCBI ORF finder. Results showed that both of Bir-01–1 and Bir-04–1 encoded five open reading frames (ORFs), while Bir-03-1 had four ORFs (Fig. 1A). Bir-01–1 encodes one nonstructural protein (NS1) and two structural proteins (VP1 and VP2), while the Bir-03-1 and Bir-04–1 encode three nonstructural proteins (NS1, NS2, and NS3) and one structural protein (VP1). The NS1 protein of these three novel parvoviruses are 640 aa, 633 aa, and 654 aa in length, respectively and possess two conserved replication initiator motifs (HuH and Yxx(x)K) and a conserved NTPase/helicase motif (Walker A, B, B’ and C) (Fig. 1B) as in other parvoviruses [1]. The BLASTp search analysis of NS1 protein showed that Bir-01–1 had the highest aa sequence identity of 99.22% with one isolate bpk075par01 (GenBank no. MT138216) from China, while had only 44.30%−74.92% aa sequence identity with other parvoviruses belonging to the genus *Aveparvovirus*. NS1 of Bir-03-1 and Bir-04–1 had the highest aa sequence identity of 66.87% and 53.09% with avian chapparvovirus (GenBank no. MN175612) reported in Brazil neognath bird and one unclassified parvoviridae (GenBank no. KY312548) isolated from fecal sample of red-crowned crane in China, respectively, while the NS1 aa sequence identity between Bir03–1 and Bir-04-1 was 42.7% (Figure S1). Based on the novel demarcation criteria proposed by Judit J. Pénzes and co-workers, viruses can be considered members of the same species if the NS1 proteins share more than 85% aa sequence identity, while NS1 proteins of members of the same genus should share at least 35–40% aa sequence identity with a coverage of >80% between any two members [1]. Therefore the Bir-01–1 belongs to a novel species of the genus *Aveparvovirus*, while the Bir-03-1 and Bir-04–1 belong to two different species of the genus *Chaphamaparvovirus*. The VP1 protein of these three strains are 675 aa, 529 aa, and 532 aa in length respectively and lack the conserved phospholipase A2 (PLA2) motif which has the associated function of virus infectivity, suggesting different mechanism for virus entry and release [7]. The BLASTp search analysis of VP1 protein showed that Bir-01–1 shared the highest aa identity of 87.41% with one isolate bpk075par01 (GenBank no. MT138216) from China. A glycine rich sequence (^163^GAGGGGGGGGVG^174^) was present in the VP1 protein of Bir-01–1 like other members of the genus *Aveparvovirus* [8]. VP1 of Bir-03-1 and Bir-04–1had the highest aa sequence identity of 76.50% and 66.34% with one isolate par083par07 (GenBank no. MW046512) from China and one isolate yc-9 (GenBank no. KY312548) isolated from fecal sample of red-crowned crane in China, respectively. No glycine rich sequences were found in the VP1 proteins of Bir-03-1 and Bir-04–1.

Phylogenetic analysis was performed based on the deduced amino acid sequences of NS and VP of parvovirus with their closest BLASTx matches in GenBank and representative members of related viral species or genera. Phylogenetic trees with 1000 bootstrap resamples of the alignment data sets were generated using the neighbor-joining method in MEGA11. Results showed that NS1 of Bir-01–1 was phylogenetically related to the strain pigeon parvovirus A (KC876004) and clustered with strains detected in wild birds (pigeon, parrot, red-crowned crane) or poultry (chicken and turkey) in China and US to form a clade that belonged to *Aveparvovirus* genus in the subfamily of *Parvovirinae*. NS1 of Bir-03-1 and Bir-04–1 clustered with strains in the newly defined subfamily *Hamparvovirinae*, of those, NS1 of Bir-03-1 was phylogenetically related to yc-9 (KY312548) which was detected in red-crowned crane in China; NS1 of Bir-04–1 clustered with BR_DF10 (MN175612) which was detected in neognath bird in Brazil (Fig. 2A). Phylogenetic trees based on VP1 of the three strains in this study were consistent with that of NS1 (Fig. 2B).

To investigate the prevalence of parvoviruses in zoo birds, three sets of primers were designed in the NS1 region of these three parvoviruses (Table 1). For screening of Bir-01–1, the forward primer: “5-CCCCTAACAGGCACAGAAGG-3”, the reverse primer: “5-GGTTGATTTGTTGCGGGACC3”; for screening of Bir-03-1, the forward primer: “5- ATCTGATGCTTGCAGACGCT −3”, the reverse primer: “5- ATTCACCACCTGTGCATCGT −3”; for screening of Bir-04–1, the forward primer: “5-CGAATGCCGTTATTGCCGAG −3”, the reverse primer: “5- TCCCCAACAGTCCCATTGTG-3” (Table 1). Results showed that 17.24% of fecal samples (10/58) were positive for Bir-01–1 which were detected in Blue peacock, Red waisted golden pheasant, Reeve’s pheasant, Silver pheasant, Cockatoos, Grey crane, and White-naped crane; 8.62% of fecal samples (5/58) were positive for Bir-03-1 which were from Blue-and-Yellow macaw, Gray parrot, and Red-crowned crane; 17.24% of fecal samples (10/58) were positive for Bir-04–1 which were detected from White crane, Gray parrot, and Red-crowned crane. It’s reported that Aveparvoviruses were widely distributed in wild birds and poultry with the prevalent rate of 12.1–78% [9]. In this study, Aveparvoviruses were detected in birds with the positive rate of 17.2% which corresponds to the previous studies. It’s reported that Chaphamaparvoviruses may be host specific. Epidemiological investigation in this study showed that four samples positive to Bir-03-1 were from *psittaciformes* order except one from *gruiformes* order; similarly, 11 samples positive to Bir-04–1 were from birds in the order *Gruiformes*. Bir04–1 and the parvovirus found in Chinese red crowned cranes *(Gruiformes)* clustered into one branch [10]. It hinted that chaphamaparvoviruses were host specific. Chaphamaparvoviruses may be pathogenic to mammals, because they were found in the inclusion body of kidney cells of mice suffering from kidney disease, and were also detected in the feces of dogs suffering from diarrhea [11, 12]. However, whether they can cause disease in birds needs further animal test or histopathological examination. Co-infection of Bir-01–1 and Bir-04–1 were only detected in one sample from Grey crane. Because no obvious clinical symptoms of zoo birds were observed during the sampling, the relationship between these three parvoviruses and disease need to be further studied.

In summary, we identified three novel parvoviruses in different zoo birds and characterized their complete genomes. The NS1 protein of these three novel parvoviruses possess conserved replication initiator and NTPase/helicase motifs. Phylogenetic analysis based on the NS1 and VP1 indicated that Bir-01–1 clustered with other strains belonging to the genus *Aveparvovirus* to form a clade, while Bir-03-1 and Bir-04–1 clustered with strains in the newly defined subfamily *Hamparvovirinae* to form two different clades. Based on the latest demarcation criteria of parvovirus, Bir-01–1 belonged to a novel species in the genus *Aveparvovirus*, while the Bir-03-1 and Bir-04–1 belong to two different species of the genus *Chaphamaparvovirus*. Epidemiologic study suggested these three viruses were prevalent in the zoo birds.

## Supplementary Material

1 **Supplementary Fig. S1**. Pairwise comparison of NS1 amino acid sequences of three parvoviruses identified in this study with the representative strains of different genera of the family *Parvoviridae*.
**Supplementary Table. S1**. The information of bird species and library included in the present study.

## Figures and Tables

**Fig.1.**
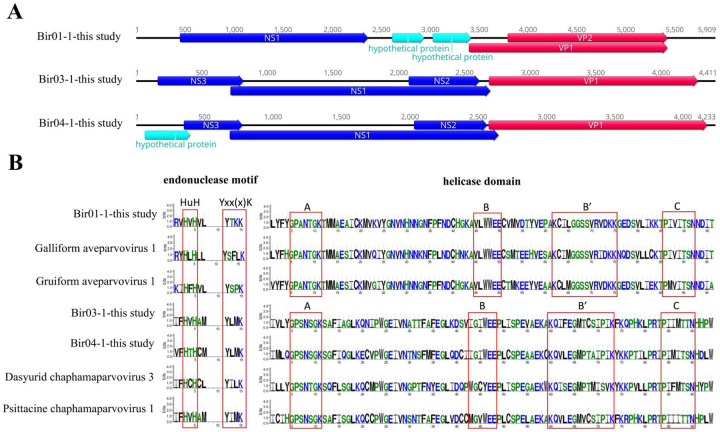
The genome structuration and conserved motifs in the NS1 region of three parvoviruses. **(A)** The genomic organization of the three parvoviruses identified in the cloacal swabs of zoo birds. Viral encoding proteins were marked. The nonstructural proteins were painted with navy blue arrow, the structural proteins were painted with red arrow, and the hypothetical proteins were painted with wathet blue arrow. **(B)** Two conserved replication initiator motifs (HuH and Yxx(x)K) and a conserved NTPase/helicase motif (Walker A, B, B’ and C) of the three parvoviruses and other reference strains were shown. The conserved motifs were marked with red frame.

**Fig.2.**
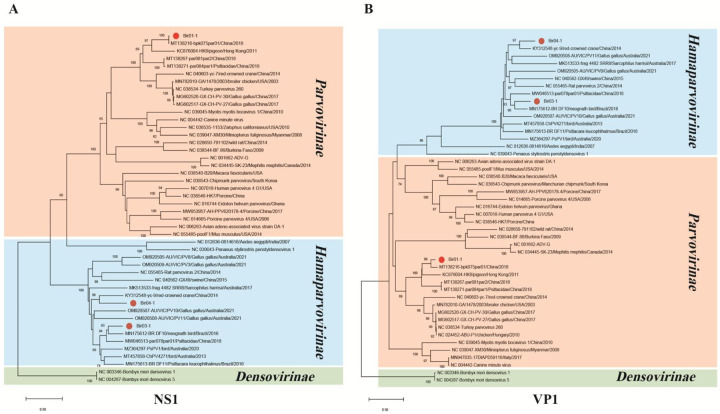
The phylogenetic analysis of three parvoviruses identified in this study. **(A)** The phylogenetic tree was constructed based on the amino acid sequences of NS1 identified in this study, and the reference strains of the subfamilies of *Parvovirinae* and *Hamaparvovirinae*. **(B)** The phylogenetic tree was constructed based on the amino acid sequences of VP1 identified in this study, and the subfamilies of *Parvovirinae* and *Hamaparvovirinae*.

**Table. 1.** Primers for PCR and detection positive rate

Virus strain	Forward primer	Reverse primer	Product size	Target gene	Positive rate
Bir01–1	5-CCCCTAACAGGCACAGAAGG-3	5-GGTTGATTTGTTGCGGGACC-3	451 bp	NS1	17.2% (10/58)
Bir03–1	5-ATCTGATGCTTGCAGACGCT-3	5-ATTCACCACCTGTGCATCGT-3	439 bp	NS1	8.6% (5/58)
Bir04–1	5-CGAATGCCGTTATTGCCGAG-3	5-TCCCCAACAGTCCCATTGTG-3	492 bp	NS1	17.2% (10/58)
