# Supplementary material for: Molecular detection and characterization of three novel parvoviruses belonging to two different subfamilies in zoo birds
Source: Res Sq. 2023 Mar 7:rs.3.rs-2593815. Preprint. [Version 1] doi: 10.21203/rs.3.rs-2593815/v1 (PMC10029121; doi:10.21203/rs.3.rs-2593815/v1)
Supplement: 1 — Supplementary Fig. S1. Pairwise comparison of NS1 amino acid sequences of three parvoviruses identified in this study with the representative strains of different genera of the family Parvoviridae. Supplementary Table. S1. The information of bird species and library included in the present study. [file NIHPPRS2593815V1-supplement-1.pdf]

Supplementary Table. S1. Information of bird species and library included in the present study.

Library number	Sample no.	Bird English name	Order name	Family name	Genus name	Species name	Library total reads	SRA no.
**Lib01**	1-3	Red-crowned crane	*Gruiformes*	*Gruidae*	*Grus*	*Grus japonensis*	2,822,810	SRX18373199
	4-5	Blue peacock	*Galliformes*	*Phasianidae*	*Pavo*	*Pavo cristatus*		
	6-7	White peacock	*Galliformes*	*Phasianidae*	*Pavo*	*Pavo cristatus*		
	8-9	Red waisted golden pheasant	*Galliformes*	*Phasianidae*	*Chrysolophus*	*Chrysolophus pictus*		
	10-11	Reeve's pheasant	*Galliformes*	*Phasianidae*	*Syrmaticus*	*Syrmaticus reevesii*		
	12-14	Silver pheasant	*Galliformes*	*Phasianidae*	*Lophura*	*Lophura nycthemera*		
**Lib02**	15	Silver pheasant	*Galliformes*	*Phasianidae*	*Lophura*	*Lophura nycthemera*	1,503,296	SRX18373200
	16-18	Amazon parrot	*Psittaciformes*	*Psittacidae*	*Amazona*	*Amazona agilis*		
	19-22	Red-and-Green macaw	*Psittaciformes*	*Psittacidae*	*Ara*	*Ara chloropterus*		

	23-27	Blue-and-Yellow macaw	*Psittaciformes*	*Psittacidae*	*Ara*	*Ara ararauna*		
	28	Gray parrot	*Psittaciformes*	*Psittacidae*	*Psittacus*	*Psittacus erithacus*		
**Lib03**	29-30	Gray parrot	*Psittaciformes*	*Psittacidae*	*Psittacus*	*Psittacus erithacus*		
	31-35	Amazona tucumana	*Psittaciformes*	*Psittacidae*	*Amazona*	*Amazona tucumana*		
	36-38	Cockatoos	*Psittaciformes*	*Cacatuidae*	Cacatua	*Cacatua galerita*		
	39-41	Grey crane	*Gruiformes*	*Gruidae*	*Grus*	*Grus grus*		
	42-43	White crane	*Gruiformes*	*Gruidae*	*Grus*	*Grus leucogeranus*	2,662,466	SRX18373201
**Lib04**	44	White crane	*Gruiformes*	*Gruidae*	*Grus*	*Grus leucogeranus*		
	45-48	Grey crane(nestling)	*Gruiformes*	*Gruidae*	*Grus*	*Grus grus*		
	49-56	White-naped crane	*Gruiformes*	*Gruidae*	*Grus*	*Grus vipio*		
	57-58	Red-crowned crane	*Gruiformes*	*Gruidae*	*Grus*	*Grus japonensis*	2,111,344	SRX18373202

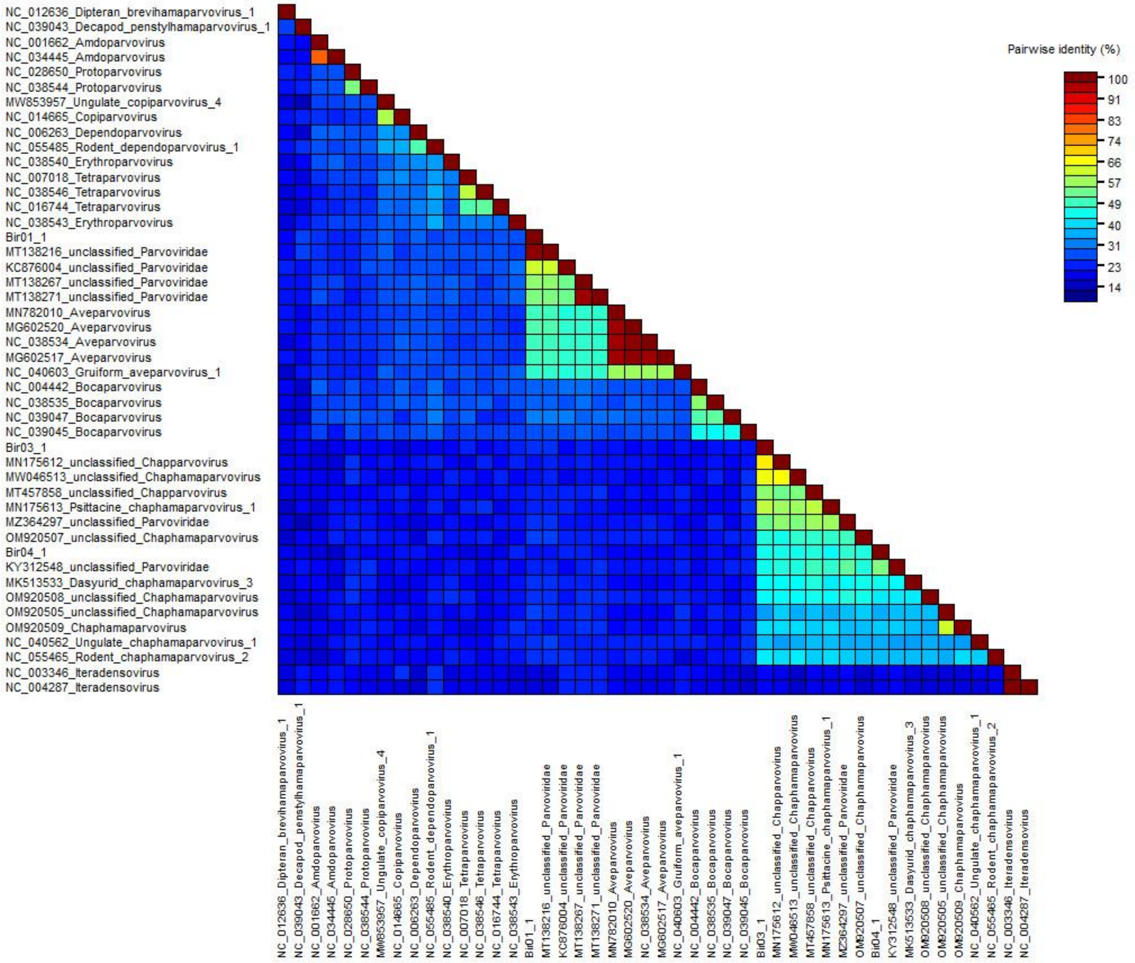

Supplementary Fig. S1. Pairwise comparison of NS1 amino acid sequences of three parvoviruses identified in this study with the representative strains of different genera of the family *Parvoviridae*.
